# Anti-*Toxoplasma* activity of *Sorghum bicolor*-derived lipophilic fractions

**DOI:** 10.1186/s13104-019-4732-z

**Published:** 2019-10-24

**Authors:** Daniel A. Abugri, Jesse M. Jaynes, William H. Witola

**Affiliations:** 1 Department of Chemistry and Department of Biology, Laboratory of Ethnomedicine, Parasitology and Drug Discovery, College of Arts and Sciences, Tuskegee University, Tuskegee, AL USA; 2 Department of Agricultural and Environmental Sciences, College of Agriculture, Environment and Nutrition Sciences, Tuskegee University, Tuskegee, AL 36088 USA; 3 Department of Pathobiology, College of Veterinary Medicine, University of Illinois, Urbana-Champaign, IL 61802 USA

**Keywords:** *Sorghum bicolor*, *Oil*-*like* extracts/fractions, Chemical composition, Anti-*Toxoplasma* activity

## Abstract

**Objective:**

*Toxoplasma gondii*, an intracellular zoonotic parasite, infects approximately a third of the world population. Current drugs for treatment of *T. gondii* infection have been challenged with ineffectiveness and adverse side effects. This necessitates development of new anti-*Toxoplasma* drugs. *Sorghum bicolor* [Moench] leaf extract has been used in African traditional medicine for the management of anemia and treatment of infectious diseases. We tested the in vitro anti-*Toxoplasma* inhibitory activity of *S. bicolor*’s oil-like crude extracts and fractions against *T. gondii* and determined their cytotoxic effects on human host cells.

**Results:**

Significant inhibitory activities against the growth of *T. gondii* tachyzoites were observed for the crude extract (IC_50_ = 3.65 µg/mL), the hexane-methanol fraction (IC_50_ = 2.74 µg/mL), and the hexane fraction (IC50 = 3.55 µg/mL) after 48 h of culture. The minimum cytotoxicity concentrations against HFF were 34.41, 16.92 and 7.23 µg/mL for crude extract, hexane-methanol and hexane fractions, respectively. The crude extract and fractions showed high antiparasitic effects with low cytotoxic effects. Further studies to determine synergistic activities and modes of action would provide impetus for the development of new toxoplasmosis drugs or nutraceuticals.

## Introduction

*Toxoplasma gondii* is an opportunistic, zoonotic, obligate intracellular coccidian protozoa affecting almost all warm-blooded and cold-blooded vertebrates [1, 2]. Globally, it is estimated that 30–50% of the human population is infected with *T. gondii* [3], with the most vulnerable being pregnant women, fetuses, infants, and immunocompromised individuals such as those with HIV-AIDS or those undergoing organ transplant or cancer therapy [4]. *T. gondii* can be acquired in various ways including ingestion of contaminated raw meat, unwashed vegetables, unpasteurized milk, contaminated water, as well as poor animal care and housing systems [5, 6]. The rate of infection and prevalence of the parasite in warm and cold-blooded vertebrates, poses medical, veterinary, food safety, public health, socio-economic and bioterrorism concerns globally [1, 7, 8]. For instance, about 22.5% of the US population aged 12 years and above have come in contact with the parasite [9]. Toxoplasmosis, the disease caused by *T. gondii*, has been reported to be the second leading cause of food-borne deaths in the US [9], and to cost the US economy about US$7.7 billion annually [10].

Current standard drugs used for treatment of *Toxoplasma gondii* infection are pyrimethamine, sulfadiazine or a combination of pyrimethamine and sulfadiazine [11–13]. However, these drugs have been reported to have limitations such as being ineffective in killing the encysted form of the parasite, and being toxic [14–16]. Hence, alternative drugs or nutraceuticals are urgently needed for treatment of toxoplasmosis. Plant-derived bioactive compounds have huge potentials as new alternatives lead-compounds for developing new generation of safe and effective drugs against *T. gondii*, partly due to documented evidence about their antimicrobial activities [17]. *Sorghum bicolor* [Moench] is a member of the Poaceae, or grass family, and is most closely allied to grass crops like sugarcane and maize*. S. bicolor* plant and its parts are rich in fatty acids, phenolics, flavonoids, peptides, iron, tannins, 3-deoxyanthocyanidins, anthocyanidins and anthocyanins, among others [18–23]. Many traditional herbal formulations have been made from its leaves and used to treat various ailments [18, 20–22]. Additionally, *S. bicolor* red leaves are used as food and drink colorant, dyes for making hats and clothing, for production of potassium hydroxide, while the flour from the seeds is used for making local cakes and beer in Nigeria and northern Ghana [20, 21]. Recently, our group discovered 3-deoxyanthocyanidins compounds that were extracted with ethanol from the *S. bicolor* to have remarkable anti-*Toxoplasma gondii* [22]. However, little is known scientifically about the identity, composition, and potential anti-parasitic effects of *S. bicolor* red leaves’ oil-like extracts and fractions. The objective of this study was to evaluate the efficacy of oil-like extracts and fractions obtained from *S. bicolor* red leaves against *T. gondii* proliferation in vitro, and to test their cytotoxicity.

## Main text

### Materials and methods

#### Plant material

*Sorghum bicolor* red leaves were obtained from Northern Ghana, West Africa as previously reported in detail [22].

#### Preparation of crude extracts and fractions

The *S. bicolor* leaf was extracted using procedures reported previously [20, 22, 24]. Briefly, about 41 g dried powdered leaves were extracted with 500 mL of 99% ethanol for 2 h at 30 °C with constant shaking at 100 rpm. The extraction was repeated three times and filtered. The crude filtrates were combined and dried using a rotatory evaporator. The solid crude extracts obtained were then washed with water, followed by *n*-hexane to obtained (12.07 g) reddish-yellow oil.

#### Fractionation of compounds from crude extract

The reddish yellow oil-like substance obtained from the *n*-hexane was passed through a size exclusion chromatography using (Biogel P 2 and P 60) with first eluded with hexane-methanol mixture (10:1). Afterwards, a pure hexane was passed through the column and the hexane fraction collected. Prior to the use of the pure hexane as eluent, other organic solvents were used but such fractions were not biologically active and thus were excluded in the chemical fingerprinting of the fractions obtained using such solvents. All the fractions collected were subjected to preparative thin layer chromatography (silica gel 60 F_254nm_, 0.2 mm: Merck) to confirm that there were differences in compound classes. Fractions with similar bands and retention factors were scrapped from the TLC plates, combined, and eluded using hexane-methanol mixture and pure hexane. All fractions were dried using a rotary evaporator. The dried crude extract and fractions were reconstituted in dimethyl sulfoxide (DMSO) for in vitro anti-parasitic and cell cytotoxicity assays.

#### Electrospray ionization mass-spectrometry analysis of lipophilic crude extract and fractions

Compounds were characterized using previously described procedure in [22]. Lipid species were identified using m/z and structural information from the compound library including those of LipidMap (www.lipidmaps.org) and Avanti Polar Lipids (www.avantilipids.com). Quantitation of the unknown lipids from biological sample extracted from plants used standard lipids with known quantity analyzed by the same method using either the ion counts or the peak area.

#### Maintenance of cell lines and parasites

Human foreskin fibroblasts (HFF) were grown in Iscove’s modified Dulbecco’s medium (IMDM) made up of 10% (v/v) fetal bovine serum (FBS), 1% (v/v) Glutamax and 1% (v/v) penicillin–streptomycin-fungizone obtained from (Life Technologies, USA) and incubated at 37 °C with 5% CO_2_ and 95% air. *T. gondii* (RH-YFP) strain tachyzoites were maintained in HFF cells for the growth inhibitory assays. To test our crude and fractions, we initially seeded HFF cells into 96 well plates using a total volume of 200 µL of IMDM medium and allowed to grow to 100% prior to use.

#### Evaluating *T. gondii* inhibitory activity

In vitro anti-parasitic activity was defined by inhibition of parasite proliferation based on parasite fluorescence intensity. The confluent HFF cells were infected with RH-YFP *T. gondii* tachyzoites (3, 500 parasites/mL). Different concentrations of lipophilic crude extract and fractions were added to the cultures in triplicate immediately after parasite inoculation. DMSO was used as a control. At 48 h interaction of parasites, drugs and cells, parasite proliferation was determined with an automated Olympus IX71 fluorescence microscope. The parasite YFP fluorescence intensity was calculated using a free ImageJ software (NIH, USA). The IC_50_ of the lipophilic crude extract and fractions against *T. gondii* and cell lines were calculated using a free Graph Pad Prism.

#### Statistical analysis

The 96 well plates were monitored for parasites relative fluorescence units per well using confocal fluorescence microscopy at 48 h of interaction with test compounds. The crude extract and oil-like fractions concentrations in microgram per milliliters were transformed into log and plotted against parasites relative fluorescence units normalized into percent. The data were then analyzed using GraphPad Prism, and the 50% inhibitory concentrations for the crude extract and oil-like fractionates against *T. gondii* and HFF cell lines were derived. The selectivity indexes (SI) for crude extract and fractions were calculated using the formula = IC_50_cells/IC_50parasites_. All data are reported as three (n = 3) independent experiments with their 95% confidence intervals. Graph Pad T-test was used to compare difference between parasites IC_50_ values and cells IC_50_ values at *P* < 0.05.

### Results

In this study, we investigated *S. bicolor* red leaves reddish-yellow oil-like crude extract and fractions anti-*Toxoplasma* activity, as well as analyzed their cytotoxicity against an HFF cell line. The total yield of the reddish-yellow oil-like crude extract obtained from *S. bicolor* red leaves was 29.44% on dry weight basis (DWB). The anti-*Toxoplasma* activity of the *S. bicolor* red leaf *n*-hexane crude extract, and those of the hexane-methanol (10:1) and *n*-hexane fractions obtained from the *S. bicolor* crude extract are presented in (Table 1). The compounds found in the crude reddish-yellow oil-like extract in significant amounts were benzoic, octanoic, nonadecanoic, linoleic, palmitic, stearic, oleic acids, arachidonic, ferulic, caffeic, quinic and jasmonic acid, as well as, luteolin, eugenol and apigenin (Table 1). Some of these compounds (e.g. benzoic, ferulic, caffeic, quinic, jasmonic acid, luteolin, and apigenin) are not usually found in lipophilic phase of plant extracts, however, we believed that their presence in the oil-like phase might have been due to incomplete evaporation of the water from the extratum.

**Table 1:** Chemical composition of the main constituents of the crude oil-like extract and its fractions obtained from dried *S. bicolor* red leaves

Compound	Hexane-methanol oil-like fraction (%)^a^	Pure hexane fraction (%)^b^	*n*-Hexane crude oil-like extract (%)^c^
Ethylformate	0.07	Tr	ni
Isobutyric acid	0.04	0.58	ni
Oxalic acid	0.05	nd	7.87
Benzoic acid	11.33	4.68	14.66
Tyrosol	1.40	2.71	1.69
Octanoic acid	ni	ni	13.95
Cinnamic acid	ni	ni	3.31
Vanillic acid	2.11	3.88	1.00
Decanoic acid	ni	nd	2.07
Caffeic acid	0.26	5.08	2.46
Quinic acid	ni	ni	2.77
Ferulic acid	ni	6.78	2.16
*n*-Hexadecanoic acid	ni	ni	3.70
Heptanoic acid	nd	ni	0.49
Myristic acid	0.05	ni	1.33
Nonadecane	0.03	ni	ni
Nonadecanoic acid	ni	ni	8.51
Palmitoleic acid	ni	ni	1.85
Indole-3-acetic acid	ni	ni	0.88
Apigenin	31.27	ni	0.75
Naringenin	2.71	0.97	0.16
Gamma-Linoleic acid	1.26	ni	0.40
Linoleic acid	1.26	0.19	1.08
Stearic acid	ni	ni	2.27
Luteolin	25.28	2.91	9.40
Gadoleic acid	nd	ni	0.91
Malvidin	nd	ni	0.62
Carsonic acid	nd	ni	0.66
Rosmanol	nd	ni	0.65
Malic acid	nd	7.86	ni
Ethylpropionate	nd	1.94	0.06
Caproic acid	nd	7.13	Tr
Alpha-thujene	nd	4.32	Tr
3,4-Dihydroxybenzoic acid	nd	4.11	Tr
Alpha-terpineol	0.06	3.10	Tr
Hydroxytyrosol	0.96	ni	Tr
Nonanoic acid	n/i	3.10	Tr
Undecylic acid	n/i	0.87	Tr
Jasmonic acid	0.74	22.93	Tr
9,10 di-Jasmonic acid	nd	1.74	Tr
Tridecylic acid	n/i	0.97	Tr
Nonane	n/i	9.88	Tr
Palmitic acid	0.88	0.78	Tr
Hesperidin	n/i	0.97	Tr
Citronellal	0.02	nd	Tr
Pelargonic acid	0.01	nd	Tr
Eugenol	3.25	nd	Tr
Coumaric acid	0.77	nd	Tr
Apigeninidin	0.05	nd	Tr
Lignoceric acid	0.80	nd	Tr
Alpha-linoleic acid	0.38	nd	Tr
Epicatechin	1.37	nd	Tr
Catechin	0.04	nd	Tr
EPA	1.78	nd	Tr
Arachidonic acid	9.57	nd	Tr
Dihomo-γ-linolenic acid	0.58	nd	Tr
Coumaric acid hexoside	0.51	nd	Tr
DHA	0.10	nd	ni
Campesterol	0.05	nd	ni
Ellagic acid hexoside	0.03	nd	ni
Ellagic acid pentoside	0.07	nd	ni
Epicatechin 3-*O*-gallate	0.07	nd	ni
Valoneic acid dilactone	0.06	nd	ni
Quercetin pentoside	0.24	nd	ni

*nd* not determined, *ni* not identified, *Tr* trace amount, ^a^ of 3.0 mg/mL, ^b^ of 6.8 mg/mL and ^c^ of 6.6 mg/mL

**Fig. 1:**
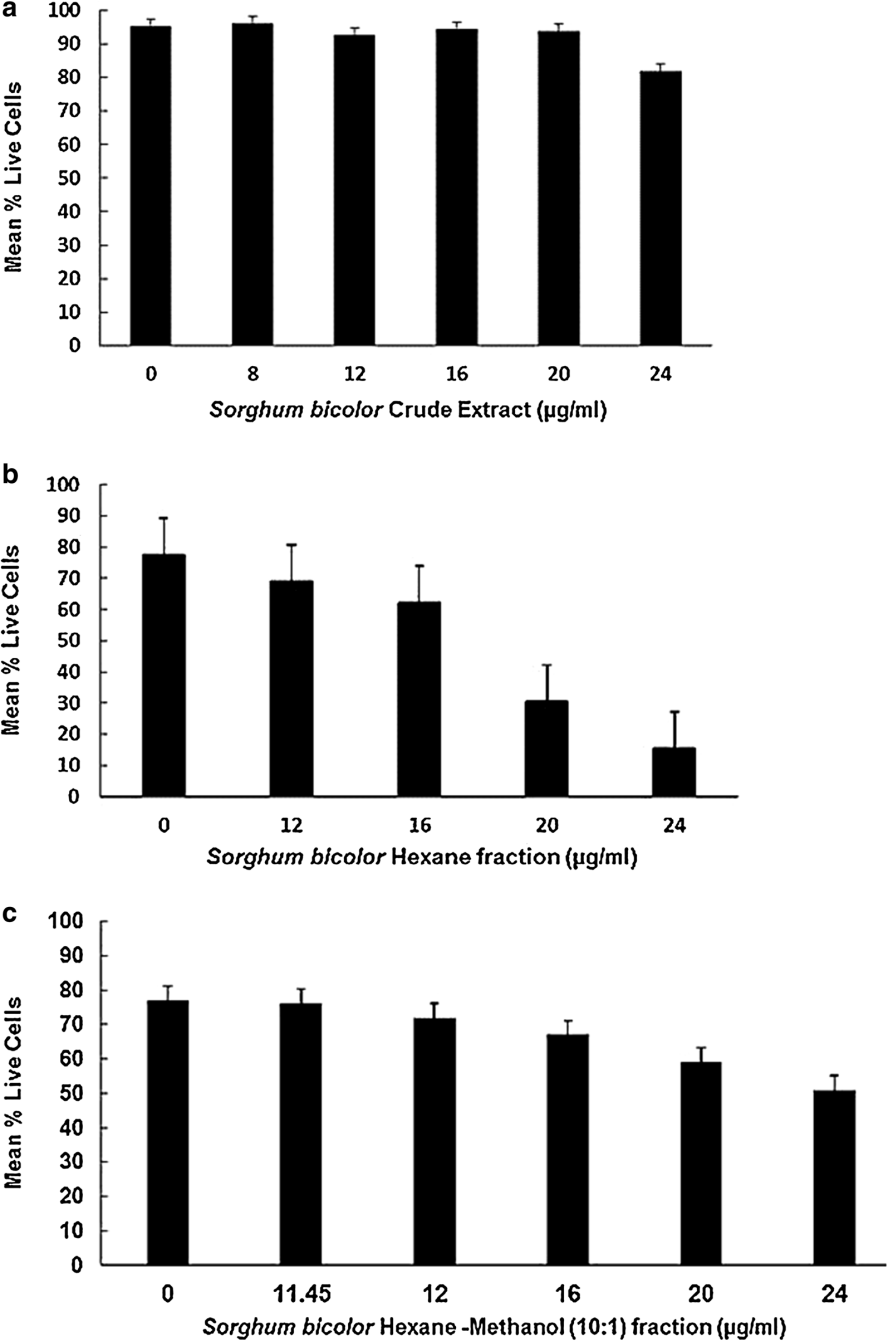
**a** Cytotoxic effects of lipophilic *S. bicolor* leaves crude extract on Human foreskin fibroblasts cells. **b** Cytotoxic effects of the hexane fraction from the lipophilic *Sorghum bicolor* extract on human foreskin fibroblast cells. **c**Cytotoxic effects of the hexane-methanol (10:1) fraction from the lipophilic *Sorghum bicolor* extract on human foreskin fibroblast cells

In our quest to establish whether the crude extract and its fractionated compounds had potent activity against *T. gondii*, we measured their minimum inhibitory concentrations against *T. gondii* tachyzoites (IC_50p_), and their cytotoxicity against HFF cells (Table 2). The crude *n*-hexane extract had significant activity against *T. gondii* with IC_50p_ value of 3.65 µg/mL, and showed an HFF cytotoxicity (IC_50c_) value of 34.41 µg/mL, which was ninefold higher than its effective concentration against parasites (Table 2). At 48 h, the IC_50p_ value (2.74 µg/mL) of the hexane-methanol fraction was lower than the value for the crude extract and the hexane fraction (Table 2), suggesting that the hexane-methanol fraction was more potently active against *T. gondii*. Importantly, the hexane-methanol fraction was less cytotoxic to HFF cells than the crude extract (*P* ˂ 0.05) (Table 2). Cytotoxic effects of the three different oil-like fractions are summarized in Fig. 1a–c. Comparatively, all the compounds tested (crude extract and the fractions) had IC_50p_ values for *T. gondii* that were significantly lower than their respective cytotoxicity IC_50_ values in the HFF cell line (*P* ˂ 0.05). Although, the hexane-methanol fraction had the lowest IC_50p_ for parasites, the selectivity index (SI) value determined for the crude extract was greater than of the hexane-methanol fraction, suggesting that the crude oil-like extract of *S. bicolor* would have broader spectrum and better potency against the parasites than the fractions (Table 2).

**Table 2:** In vitro anti-*Toxoplasma gondii* growth inhibitory effect (µg/mL), cytotoxic activity (µg/mL), and selective indexes for *S. bicolor* red leaf oil-like extract and fractions

Compound	CC_50_ (95% CI)	IC_50_ (95% CI)	SI
*S. bicolor n*-hexane crude extract	34.41 (31.62–37.45)	3.65 (2.07–6.45)	9.43
Hexane-methanol (10:1) fraction of crude extract	16.92 (14.25–20.10)	2.74 (0.71–10.54)	6.18
Hexane fraction of crude extract	7.23 (3.24–16.14)	3.55 (0.73–17.20)	2.04

*95% CI* 95% confidence interval for means of triplicate independent assays, *CC*_*50*_ concentration of oil-like extract that caused 50% reduction in viability of human foreskin fibroblasts, *IC*_*50*_ minimum inhibitory concentration of crude and fractionated oil-like substance that inhibits 50% *T. gondii* tachyzoite growth, *SI* selectivity index

### Discussion

The non-availability of safe and effective vaccine or drugs to completely eradicate neglected infectious diseases is still a major global challenge, partly because of the emerging drug-resistant strains of pathogens, coupled with toxicity and hypersensitivity of some current drugs. Hence, there is an urgent need to develop new effective and safe antitoxoplasmosis drugs from ethnomedicinal plants. Here, we investigated the inhibitory activity of oil-like extracts from dried *S. bicolor* red leaf against *T. gondii* tachyzoites. The *S. bicolor* oil-like crude extracts and fractions were found to significantly inhibit *T. gondii* tachyzoites growth in vitro. Generally, the cytotoxicity levels of the different fractions were twofold (*n* hexane fraction), sixfold (*n* hexane-methanol fraction) and ninefold (crude extract) greater than the *T. gondii* effective inhibitory concentrations. These observations suggest that, despite having had potent activity against *T. gondii*, *S. bicolor* oil-like crude and fractions may not pose adverse side effects against mammalian cells. This unique activity of *S. bicolor* red leaf oil-like crude extracts and fractions could be partly attributed to the presence of benzoic, octanoic, nonadecanoic, linoleic, palmitic, stearic, oleic, arachiodonic, ferulic, caffeic acid, quinic and jasmonic acid, as well as luteolin, apigenin and eugenol. Studies of antibacterial, antifungal, antiviral and antiprotozoal activities of some essential oils have been reported [25–27]. However, the compounds composition in the extracts reported earlier are not similar to the ones found in the present study. Importantly, the oil-like extracts and fractions obtained from the *S. bicolor* leaf were observed to have high potent anti-*T. gondii* activity than other essential oils antiprotozoal studies reported [26–28]. This could be attributed to the presence of the 3-deoxyanthocyanidin compounds that have been previously reported from the sorghum leaf to have remarkable antiparasitic activity [22]. In comparison, the IC_50_ values against *T. gondii* for the various oil-like fractions reported in this paper were lower than some of those reported by several authors in different medicinal plant extracts [27–29].

Nevertheless, further studies would be important to determine the cytotoxic effects of the *S. bicolor* crude oil-like extract and its fractions in additional mammalian cell lines. Notwithstanding, the *S. bicolor* oil-like extracts and fractions investigated in this study exhibited strong anti-*Toxoplasma gondii* activity with low cytotoxic effects against a human cell line (HFF). Eugenol, a compound we found in significant amounts in the *S. bicolor* extract has been shown to have anti-*Giardia*, antibacterial, antifungal and antioxidant properties, as well as curative effects in skin, prostate and melanoma cancers [30, 31]. On the other hand, luteolin and apigenin have also been reported to have anticancer, antibacterial and anti-leishmania activities [32, 33]. Tasdemir et al. [33], also attested that phenolic acids such as coumaric acid, caffeic acid, ferulic acid and cinnamic acid have effective in vitro leishmanicidal activity.

Taken together, immunocompromised persons such as cancer patients may derive multiple benefits from *S. bicolor* essential oils in fighting cancer and any *T. gondii* opportunistic coinfections.

## Limitations

The *S. bicolor* oil-like crude extracts and fractions has potency against the tachyzoite stage, and it will be interesting to determine if it may also have activity against the encysted bradyzoite stage of *T. gondii*. It will also be important to study the *S. bicolor* oil-like crude extracts and fractions further in order to identify the individual compounds responsible for the anti-*T. gondii* activity and determine their mechanism of action individually or in combinations for the development of novel inhibitors against *T. gondii* and other pathogens.

## Data Availability

The datasets used and/or analyzed during the current study are available from the corresponding author on reasonable request.

## Abbreviations

DMSO: dimethyl sulfoxide
DWB: dry weight basis
FBS: fetal bovine serum
HFF: human foreskin fibroblasts
HIV-AIDS: human immunodeficiency virus and acquired immune deficiency syndrome
IC_50p_: minimum inhibitory concentrations against *T. gondii* tachyzoites
IC_50c_: the minimum cytotoxicity concentrations against HFF
IMDM: Iscove’s modified Dulbecco’s medium
TLC: thin layer chromatography
RH-YFP: yellow fluorescent protein
SI: selectivity index
NIH: National Institute of Health
US: United States
